# Debates in allergy medicine: specific immunotherapy efficiency in children with atopic dermatitis

**DOI:** 10.1186/s40413-016-0106-3

**Published:** 2016-04-18

**Authors:** Tatiana A. Slavyanakaya, Vladislava V. Derkach, Revaz I. Sepiashvili

**Affiliations:** People’s Friendship University of Russia, Moscow, Moscow region Russia; Institute of Immunophysiology, Moscow, Russia; Pacific State Medical University, Vladivostok, Russia

**Keywords:** Allergen specific immunotherapy, Subcutaneous specific immunotherapy, Sublingual specific immunotherapy, Aeroallergen, Atopic dermatitis, Children, Clinical efficacy

## Abstract

Allergen specific immunotherapy (AIT) has been the only pathogenetically relevant treatment of IgE-mediated allergic diseases (ADs) for many years. The use of AIT for atopic dermatitis (AD) treatment is dubious and has both followers and opponents. The improvement of subcutaneous AIT (SCIT) and introduction of Sublingual immunotherapy (SLIT) gives prospects of their application both for adults and children suffering from AD. This review presents results of scientific research, system and meta-analyses that confirm the clinical efficacy of AIT for children with AD who has the sensitization to allergens of house dust mite, grass and plant pollen suffering from co-occurring respiratory ADs and with moderate and severe course of allergic AD. There have been analyzed the most advanced achievements in AIT studies as well as there have been specified the unmet needs in AD. The preliminary diagnostics of IgE-mediated AD and pathophysiological disorders, including immune ones, will allow a doctor to develop appropriate comprehensive treatment algorithm for children’s AD aimed at its correction. The including of AIT to the children’s comprehensive therapy program is reasonable only if AD has the allergic form. It is necessary better to design the randomized research studies and to acquire extended clinical practice in children with AD. Use of the successes of molecular-based allergy diagnostics will help to optimize and personalize the process of selecting the necessary allergens to determine the most appropriate vaccines for children considering the results of the allergen component diagnostics. The strategy of treatment of children with AD in future will be based on individual target therapy.

## Background

Atopic dermatitis (AD)—is a chronic inflammatory allergic disease (ADs) of the skin with multifactorial pathogenesis. Its development proceeds against the background of various and interdependent genetical, ecological, immunological, psychological, biochemical and other pathological processes, the most important of which is dysfunction of skin barrier [28, 44, 50, 79, 84, 96]. The skin barrier protective dysfunction causes the acceleration of secondary infection overlay and extraneous antigens penetration through damaged corneal layer. The prevalence of adults’ and children’s AD is steadily increasing all over the world and now reaches over 1–20 % (approximately 20 % for children and 1–3 % for adults) [28, 58, 72, 85].

For the last twenty years in many countries there have been fixed the increase of prevalence of the disease [29, 67, 86], as well as the increase of severe and complicated AD incidence with permanently relapsing course that is resistant to basic traditional therapy (BT) [10, 59, 77, 78, 80]. According to the references infectious complications occur in 25–34 % of children suffering from AD [8, 41, 90, 95]. Acquiring the chronic course with frequent recurrence the disease keeps its clinical features for many years. The Severe AD sharply reduces the patient’s and his family’s quality of life [29, 33, 77, 78, 80]. Allergic bronchial asthma (BA), food allergy, pollen allergy and/or allergic rhinitis (AR) develop later in 40–50 % of children suffering AD. Subsequently, 40–50 % of children suffering from AD, have BA, food allergy, pollen allergy and/or AR evolved.

## Atopic dermatitis and allergens

Ethiopathogenetic reasons for AD are still the subject of wide discussion. Most of the authors mark the critical pathogenetic role of IgE-mediated mechanisms of hypersensitivity in relation to many foods, domestic and/or environmental allergens. The Immunologic Triggers of AD are Foods, Aeroallegens, Staphylococcus aureus, Viral Infection, Autoallegens, Fungi. Many patients with AD are sensitized to seasonal and perennial aeroallergens, which can play a key role in activation or retention of the skin disease occurrence. However, it is rather problematic to obviate the pollen allergens—aeroallergens (house dust mite, grass and plant pollen).

Among the most important etiological factors in AD development are allergens of house dust mites and their waste products (HDMA). At the moment, there have been distinguished 24 HDMA, representing the families: Pyroglyphidae mainly Dermatophagoides pteronyssinus, D. farinae, and Euroglyphus maynei), Glycyphagidae (e.g. Lepidoglyphus destructor, Blomia sp.) and Acaridae (e.g. Tyrophagus putrescentiae, Acarus siro) [42, 88, 89]. These allergens are responsible for allergic reactions in diseases such as AR, BA and AD. It is determined that approximately 5 % of people are sensitive to house dust mite allergens (HDMA). Due to high enzymatic activity HDMA are able to penetrate through the damaged epidermal skin barrier of patients with AD to get the access to immune cells. HDMA cause allergic response both of immediate and delayed types that leads to worsening of AD [13]. Double-blind controlled research has proved the HDMA role in children and adults’ AD initiation [40, 87].

Gutgesell et al. [40] conducted a randomized research in studying the HDMA role in the initiation of AD and weighting its clinical manifestations, which included 20 adult patients with moderate and severe forms of AD. In the group of patients with active treatment, special measures were conducted preventing the penetration of HDMA, but the control group did not receive it. The severity of the disease was judged every 2 months throughout the year according to the established SCORAD system, eosinophil cationic protein (ECP) in serum in ELISA and quantitative use of topical steroids. In addition, weekly on a visual analogue scale the patients themselves rated daily itching and itching, inducing insomnia. Some of the patients in the active treatment group reported the reduction of itching, inducing insomnia, however, the statistically significant differences between the groups were not revealed. In adult patients with AD it has been shown that the reduction of the HDMA impact during the year improves some clinical symptoms of the disease, but does not influence on the activity of the disease. This research has confirmed the important role of HDMA in the development and maintenance of the AD symptoms. At the same time HDMA and other aeroallergens are successfully being used as potential preventive and therapeutic variant for AIT in treatment of patients with AD [5, 11, 13, 26, 27, 46, 54, 57, 60].

Deep insight of immunological disorders contributing to the AD pathogenesis has caused the increase of interest in applying of AIT according to this disease. AIT applying in case of AD requires thorough discussion and analysis of available clinical data to determine with certainty its positive or negative effect on the disease course. AIT in case of AD is still a subject of debates that take place in sessions of many scientific forums where scientists and clinicians express their opinions, discuss and provide evidences of the efficacy of this method or argue to restrict its use.

## Efficacy of Subcutaneous AIT in children with AD

Reports on AD treatment, particular in children, appear regularly in world’s literature (Table 1). First encouraging randomized studies on applying SCIT for treatment of children and adults with AD were published in 1970s. In 1974, Kaufman и Roth [45] carried out the quasi-randomized controlled study among 52 adults and children with AD. In total only 26 patients completed the experiment with SCIT that lasted for 2 years. In the process, the significant improvement was noticed in 81 % of cases in the SCIT group compared to 40 % in placebo group.

**Table 1:** Clinical studies of SCIT/SLIT efficacy in AD

Authors	Year of research	Country	Study design	The number of patients (treatment/control)	Category of the patients (Age)	The type of allergen	Route of administration	The total duration of the study (in months)	Clinical efficacy (the opinion of the Clinician -C/patient-P) Treatment/control (in %)	Reference number
Kaufman H.S., Roth H.L.	1974	US	qRCT DB PC	52 (26/26)	children/adults (2–47)	animal dander, HDM molds, pollen	SCIT	24	C(+)	45
									81,3/40	
Warner J.O. et al.	1978	UK	RCT DB PC	20 (9/11)	children (5–14)	HDM	SCIT	12	P(+)	92
									77,8/27,3	
Ring J.	1982	Germany	RCT DB PC	2 (1/1)	children (10)	Grass pollen	SCIT	24	C(+)	71
									SCORAD improvement from score 30→10/26→21	
Glover M.T., Atherton D.J.	1992	UK	RCT DB PC	24 (13/11)	children (5–16)	HDM	SCIT	8 -13,5	P(+)	39
									61,5/81,8	
Galli E. et al.	1994	Italy	RCT DB PC	60 (NM)	children (0,5-12)	HDM	SCIT	Till 36 (mean duration 18.7)	C(+)	38
									Group A (AD+AR/BA)/Group B&Group C (AD) Improvement of skin lesions: 81/63/61	
Silny M., Czarnecka- Operacz W.	2006	Poland	RCT DB PC	20 (10/10)	children/adults (5–40)	dander, HDM pollen (adsorbed with aluminum hydroxide)	SCIT	12	C(+)	76
									80/10 on the base of W-АЗС index, specific IgE (р<0,001)	
Silny W. et al.	2005	Poland	CT	68 (36- HDM+ 12-Pollen/20-BT***)	children/teenagers	HDM Pollen	SCIT	36	C(+)	73-75
									Significant clinical efficacy W-АЗС index > BT (р <0,001)	
Czarnecka- Operacz M, Silny W.	2006	Poland	NM	66	NM (subgroup with different age)	HDM/grass pollen/grass + mugwort pollen	SCIT	48	C(+)	25
									Significant clinical efficacy with IgE-mediated airborne allergy	
				37(14/17/6)/ 29						
Bussmann C. et al.	2006 (2007)	Germany	pilot study (systematic overview)	25	NM	HDM	SCIT	4	C(+)	13,14
									SCORAD, specific IgE, IgG4, IL-10	
Slavyanskaya T. et al.,	2013-2015	Russia	CT	350	children/teenagers (3–18)	HDM	SCIT	≥36	C(+)	29-35, 77,78, 80-83
Derkach V. et al.				300/50 (moderate-severe AD)					QOL (scores) 11.32→ in 3 times decreased (р<0,001), SCORAD	
									42,57→5,19, specific IgE, IL-4, IL-10	

Note: *SCIT* subcutaneous immunotherapy, *SLIT* sublingual immunotherapy, *AD* atopic dermatitis, *AR* allergic rhinitis, *BA* allergic bronchial asthma, *HDM* house-dust mite, *qRCT* quasi-randomized controlled trial, *RCT DB PC* randomized controlled trial double-blind placebo-controlled, *CT* clinical trial, *NM* not mentioned, *SCORAD* severity scoring of atopic dermatitis, *QOL* quality of life, *C (+)* presence, by clinician, *P (+)* presence, by patient

In 1978, Warner et al. [92] conducted the double blind randomized placebo-controlled research of children with BA and 20 children who additionally have signs of atopic allergy. Upon the 1 year of treatment with SCIT, in the patients’ and their parents’ opinion, there was noticed the subjective improvement of eczema in active treatment group (77.8 %), compared to minimal improvement in placebo group (27.3 %).

In 1980–90s results of double blind placebo-controlled studies were published where Ring [71] and Glover et al. [43], showed the efficacy of SCIT applying for AD treatment.

Thus, the experiment was carried out on treatment of identical twins suffering from atopic eczema (with spring and summer exacerbations), one of whom got SCIT with grass pollen allergens and another—got placebo. Patient who got immunotherapy showed significant improvement of clinical features and reduction of serum IgE levels (Ring [82]).

Glover et al. [39] in their research of applying SCIT with HDMA in treatment of 24 children with AD revealed the statistically significant change of clinical efficacy in comparison with placebo only if it had been applied for more than one year. That demonstrated the necessity of carrying out such treatment for a long time to reach the clinical effect.

In 2006 EAACI/AAAAI in joint document (PRACTALL Consensus Report) [93], dedicated to AD diagnostics and treatment brought into focus that further well-controlled studies are required to determine future role of immunotherapy for children and adults with AD.

And already in 2006 Silny et al. [76] presented their study with the results of double blind placebo-controlled research of patients with AD. For the SCIT efficacy estimation in AD control, they used both clinical and immunological criteria. During 12 months, 20 patients aged between 5 and 40 years old with AD and sensitization to HDMA or grass pollen allergens got SCIT with medications of allergens adsorbed with aluminum hydroxide. Clinical efficacy of treatment (the extent and severity of patients’ with AD skin inflammation against itching and sleep disturbance) was assessed by W-АЗС index. Immunological parameters included the research of levels of general and specific IgE in the blood serum, ECP, SIL-2R, IFN--γ, IL-4, IL-5 and IL-10. It was shown that after 12 months of therapy there was proven improvement of clinical features in the group of patients who had got SCIT compared with placebo group (*р* < 0.001). The level of serum specific IgE in the SCIT group had a tendency to decrease, while in the placebo group it had a tendency to increase. The concentration of serum immunological parameters including ECP, SIL-2R, IFN-γ, IL-4, IL-5 and IL-10, which were analyzed before and after treatment, didn’t show the significant difference. Judging by the significant improvement of clinical index and reliable decrease of specific IgE level, SCIT turned out to be quite effective method of IgE-mediated AD treatment.

Other series of studies (not blind, placebo-controlled) was made by Silny et al. [73–75] on 36 children and teenagers with AD. 24 patients sensitive to HDMA and 12 patients sensitive to grass pollen allergens were given SCIT with respective allergens for 3 years. The control group included 20 AD patients with similar IgE-mediated AD, who were given BT. The clinical efficacy of specific immunotherapy based on the W-АЗС index was much higher than that of BT (*р* <0.001). By the end of the treatment course there was determined clear reliable difference in the values of the studied immunological parameters between experimental and control group (concentration of general and specific IgE, ECP, SIL-2R, IL-4, IL-5). SCIT turned out to be effective in case of its long-time applying to children with Ig-E-mediated AD.

Of note, conducting randomized research in children is very difficult. However, over the last 20 years a lot of isolated open pilot studies were made in this field. They confirmed the clinical efficacy of SCIT with aeroallergens (HDMA, grass, birch, sage pollen allergens) with long-term (from 1 to 3 years) treatment of children and adults with moderate and severe AD or AD developing on the background of AR and BA, where SCIT was used as an additional therapeutic approach to BT [13, 14, 23, 25–27, 49, 53, 55, 56, 72, 74, 91, 94]. Good results were obtained in all studies that allowed the authors to consider SCIT to be quite prospective method of skin inflammation treatment and really effective and safe alternative method of treatment for patients with AD having IgE-mediated allergy to aeroallergens.

Our experience of using SCIT in children 5–18 years old with moderate and severe AD and sensitization to HDMA also has showed the positive results [29–31, 34, 35, 77, 78, 80, 82, 83]. In the plan of treatment, except BT and SCIT, additionally was included immunomodulator [31–33, 81, 82]. In the group of children with AD who has used SCIT for 3 years minimum a significant reduction of SCORAD-index was recorded as well as the decreased number of exacerbations, the reduced amount of BT without AD symptoms augmentation and inpatient care. They have also showed the improvement of their lives quality and long-term effect of treatment (period of observation is over 5 years). Besides SCIT was pharmaco-economic efficiency.

## Efficacy of Sublingual AIT in children with AD

Sublingual immunotherapy (SLIT) was introduced as safer variant of SCIT [12, 18–20, 22, 23, 63, 66] to solve the problem of adverse reactions that are rare but possible with traditional SCIT. SLIT treatment is well tolerated, adverse reactions are local; mostly the reactions are in mouth cavity or gastrointestinal tract. System reactions related to skin, respiratory tract or anaphylaxis are extremely rare [37]. SLIT is much safer than SCIT and no evidences of anaphylaxis were registered after the infusion to different patients with AD more than 500 million doses. SLIT is significant step forward and is particularly suitable for pediatric patients.

However, to assess the clinical effectiveness there was made a number of clinical studies, including randomized, placebo-controlled. Despite the fact that number of AD studies is not enough, however the results obtained by the authors are promising (Table 2).

**Table 2:** Summary characteristics of the clinical efficacy of SLIT in AD

Authors	Year of research	Country	Study design	The number of patients (treatment/control)	Category of the patients (Age)	The type of allergen	Route of administration	The total duration of the study (in months)	Clinical efficacy (the opinion of the Clinician -C/patient-P) Treatment/control (in %)	Reference number
Petrova S.I. et al.	2006	Russia	RCT DB PC	99 (28-SLIT/39-placebo/32-BT)	teenagers/adult	HDM	SLIT	NM	C(+)	68
									Improvement in the SLIT group	
Pajno G.B. et al.	2007	Italy	RCT DB PC	56 (28/28)	children (5–16)	HDM	SLIT	18	C(+)	60
									Improvement in mild-moderate forms after 9 months of treatment. SCORAD was significant (P = .025)	
Di Rienzo V. et al.	2014	Italy	Multicentric RCT open, parallel-group study	NM	children (5–18)	HDM	SLIT	18	C(+)	36
									Effectively in children with AD	
Qin Y.E. et al.	2014	Chinese	RCT DB PC	107 (58/49)	NM	HDM	SLIT	12	C(+)	70
									77.78/53.85 (P < 0.05)	
Cadario G. et al.	2007	Italy	pilot study	86 (53 females and 33 males)	children/adults (3–60)	HDM	SLIT	12	C(+)	16
									SCORAD Improvement from score 43,3→23,7 Reduces the SCORAD	

Note: *SLIT* sublingual immunotherapy, *AD* atopic dermatitis, *AR* allergic rhinitis, *HDM* house-dust mite, *RCT DB PC* randomized controlled trial double-blind placebo-controlled, *CT* clinical trial, *NM* not mentioned, *SCORAD* severity scoring of atopic dermatitis, *C (+)* presence, by clinician

In 1994 Galli et al. [38] published first placebo-controlled study with the use of oral allergen-specific therapy in children with AD sensitive to HDMA. The study involved 60 children who were divided into 3 groups: children with AD and children with AD in combination with BA/AR. A group of children with AD was divided into 2 parts: one of which was a control group and was given placebo. Two other groups got active treatment. In total, clinical studies were conducted during 3 years. And thought the study did not reveal significant difference in the clinical assessment of dermatological changes in the groups, the authors made a conclusion that oral therapy with HDMA extracts occurs without any adverse reactions and doesn’t affect the natural course of AD.

In 2007 Pajno G.B. et al. [60] conducted a double blind randomized placebo-controlled study using SLIT with HDMA in 56 children with AD between 5–16 years old with SCORAD index > 7. 28 children took SLIT for 18 months in addition to standard therapy. It is important to note that in the group of children treated with SLIT, SCORAD index began to decline abruptly after 9 months of treatment and need for drugs for the treatment of AD in this group was reduced significantly compared with placebo group. A positive result of this therapy was noticed only in the patients with mild, moderate AD, but not with severe form of AD. The authors made a conclusion that SLIT can be an additional therapeutic tool for the treatment of allergic AD in properly selected, for such method, children.

In an open, uncontrolled, non-randomized pilot study Cadario et al. [16], conducted on 86 adults and children aged 3–60 years with mild and moderate IgE-mediated AD, studied the clinical efficacy (SCORAD) and safety of SLIT with HDMA. The use within 12 months of SLIT with HDMA extract in the patients with mild and moderate AD was effective and well tolerated. After a year of SLIT treatment decrease of SCORAD index was noted and the gradual reduction of concomitant therapy with actual corticosteroids or immunosuppressors.

Larenas-Linnemann [47] presented an analytical review of the studies on the use of modified, modern variants of AIT – SLIT and oral AIT which are most interesting for use in pediatric practice. Analysis of the result of tests carried out on adults and children showed that with a high degree of certainty it can be proven that high doses of SLIT, in its daily conduct, reduce both ADs symptoms and amount of drugs required for treatment. Low doses of SLIT reduce the development of new sensibilizations and in mild and moderate form of AD course decrease the symptoms of the disease.

The Systematic review and meta-analysis of 8 studies (6 – using SCIT and 2 with SLIT) on the AIT efficacy in 385 patients (adults and children) with AD sensitive mainly to HDMA Bae et al. [9] revealed that the use of aIT is justified in case of severe uncontrolled AD. In general, despite the dichotomous results presented in the studies, the meta-analysis provided moderate quality evidence of AIT efficacy in AD. However, these conclusions were based on analysis of a small number of randomized controlled studies with significant heterogeneity in research, which did not allow to make final conclusion about the efficacy of AIT in AD.

Pleskovic N. et al. [69] when comparing SCIT and SLIT in children showed similar clinical efficacy, but SLIT was a safer therapy. SLIT is confidently recommended for use in pediatric practice for patients suffering from AR or ARC for the environmental allergens. The use of SLIT as an alternative treatment for pediatric patients with AD is under development.

In 2014 Di Rienzo et al. [36] conducted a multicenter randomized study on the use within 72 weeks of SLIT with HDMA in children 5–18 years old with AD associated/not associated with allergic respiratory diseases and came to the conclusion that immunotherapy was efficient for children with AD.

Arkwright et al. [7] in their study showed an example of SLIT treatment of 7 years old child with severe AD and high concentration of IgE to HDMA. It was found that SLIT didn’t lead to an improvement of AD course but had a beneficial impact on the girl’s collateral AR.

Choi et al. [21] in their work has published the results of using for 1 year SLIT with HDMA treatment in 11 years old boy with recurrent severe AD from which he had suffered for several years. After first 6 months of the treatment the significant clinical improvement of his dermatitis was noticed as well as the reduction of SCORAD to 15 and further mild improvement after 12 months (SCORAD 12). The condition of the affected skin which was resistant to traditional therapy improved significantly. The child continued SLIT treatment with excellent tolerance to HDMA. Based on that data the authors make a conclusion that SLIT with long-term use can be a safe alternative treatment of recurrent allergic AD.

The safety profile and efficacy of SLIT in children with mild and moderate HDMA-induced AD, pointed out that SLIT can be considered as a real option of treatment of patients with ADs including AD [19, 43, 61, 62, 64–66, 68, 70]. By 2014 the evidences of SLIT efficacy in treatment of different AD were already based on the results of 77 clinical studies and 7 meta-analyses [62].

## AIT for AD the optimism and the issues

Despite the fact that there is a great progress in introduction of AIT there are still a lot of questions that remain unanswered [24, 48]. One of them is the use of AIT in AD especially in children that causes wide discussions in the scientific circles [48].

Usually AIT in children with AD is not used as a first line therapy, so for infants and babies with positive family anamnesis of AD, AIT should be considered as an option for the secondary prevention from atopic diseases [17]. If children have AD on the background of AR or BA with sensitization to aeroallergens, AIT can be considered as a therapeutic agent to reduce the progression of concomitant respiratory pathology as well as to prevent the sensitization to unrelated allergens (SCIT -[25, 38, 74, 92]; SLIT – [36, 51, 52]).

The clinical efficacy of AIT for patients with AD, especially for children, is controversial. The amount of studies in this area for adults using various variants of AIT in AD significantly exceeds the ones conducted for children, and question of possibility of extrapolation of adult data to children’s ones remains open, though similar pathogenic mechanisms of development of allergic inflammation lay on the basis of development of Ig-E-mediated ADs. However, the strength of immune response may vary and depends on individual characteristics of a child.

Conducted all over the world for the past 30 years numerous both open, pilot and double, blind, placebo-controlled studies, review of systematic and meta-analyses, have allowed to identify the conditions under which AIT may be useful in AD. At the moment it can be stated that AIT, in compliance with all necessary conditions for its implementation, can be really effective and safe treatment option for a specific group of patients with IgE-mediated AD with sensitization to aeroallergens (in particular, to HDMA, grass and plants pollen allergens) or in severe uncontrolled form of AD when the benefits of using AIT exceed the risk of adverse effects occurrence.

In Table 3 shows the comparative clinical effectiveness of SCIT vs SLIT in AD, where the evidences that have already been confirmed in clinical trials are listed. Also, there are the questions mentioned that still remain unanswered.

**Table 3:** Comparative effectiveness of different methods of AIT in AD in children (SLIT vs SCIT)

CLINICAL EFFICACY	AIT (route of administration)	
	SCIT	SLIT
WHAT IS KNOWN?	(+) in Ig-E mediated AD	(+) in Ig-E mediated AD
	(+) in AD in combination with respiratory allergies (AR and BA)	(+) in AD in combination with respiratory allergies (AR and BA)
	(+) to mono aeroallergens (HDM, pollen of grass and plants)	(+) to mono aeroallergens (HDM, pollen of grass and plants)
	(+) to a mixture of aeroallergens (HDM, pollen of grass and plants)	(−)
	Have been identified effective and ineffective doses for many allergens	Have been identified the appropriate dosages for SLIT tablets for aeroallergens (allergens of grass, ragweed and house dust mites)
	Possibly greater efficacy (at least first year)	(−)
	Effective when using a mixture of multiple aeroallergens	(−) to a mixture of aeroallergens (HDM, pollen of grass and plants)
	The main duration of therapy is 3–5 years	The optimal duration of therapy is 3-4 years
	Prevention of new sensitization and progression of respiratory allergies (AR and allergic BA)	Prevention of new sensitization and progression of allergic BA
	Lasting effect after termination of treatment	Lasting effect after termination of treatment
	May cause local and systemic reactions	Improved safety compared to SCIT (mostly, temporary local reactions)
	More systemic reactions	
	Inconvenient in use (requires special conditions: a trained expert, equipped rooms and conducting in outpatient conditions, patient’s observation)	More convenient in use compared to SCIT (can be applied at home)
WHAT REMAINS UNANSWERED?	The comparative efficacy compared to SLIT	The comparative efficacy compared to SCIT. Possibly less effective (at least first year)
	Possibly more effective (at least first year)	
		Optimal dosing of SLIT in drops Optimal dosing regimens are defined only for grass, ragweed and HDM tablets
		The possibility of using mixtures of multiple unrelated allergens
		Multiple allergen mixes can be less effective.

Note: *AIT* allergen specific immunotherapy, *SCIT* subcutaneous immunotherapy, *SLIT* sublingual immunotherapy, *AD* atopic dermatitis, *AR* allergic rhinitis, *BA* allergic bronchial asthma, *HDM* house-dust mite, *(+)* clinical efficacy, *(−)* clinically ineffective or poorly understood

For the AIT conduction there should be a strict selection of patients with prior research in the area to confirm the diagnosis of IgE-mediated disease. The preliminary diagnostics of immunopathogenetic phenotype of AD and pathophysiological disorders, including immune, will allow a doctor to develop the appropriate algorithm of complex treatment of AD in children, aimed at their correction. The individual approach is required to adjust allergen dose for AIT taking into account pathophysiological and immune changes, the degree of patient’s allergization, defined by skin tests, which can be further also used to assess the clinical efficacy of AIT in children in the treatment process. A very important factor is setting an age limit to start the immunotherapy (often AIT is recommended to start treating in children of minimum 5 years old). The use of AIT must be justified and conducted under a highly qualified doctor’s supervision, regardless of the ways of infusion the allergens.

Nowadays there is a lot of documents regulating the conduct of AIT in patients with respiratory allergy, including children, however there are still no clear recommendations regarding AD. All guidelines mark AD as a disease that requires further careful research. The unmet needs of AIT treatment in children with AD coincide in many ways with those for children with AR and BA, which were published by EAACI in 2012 [17]. In AD, however, there is no clearly defined methodological base where all the guiding principles of AIT conduct could be registered, where could be taken into account the optimal doses and schemes, safety of methods and possible adverse effects, the rules of first aid, indications and contraindications, impact on life quality, pharmaco-economic aspects, etc.

Recently published randomized results of the combined use of the two methods (SCIT and SLIT) in children for the treatment of BA and AR [60] have shown the best clinical efficacy compared to the use of mono method (separately SCIT or SLIT) or BT. The new interpretation of AIT method has proved to be the most promising, as it has included the abilities of both immunotherapy methods: SCIT has quickly activated the tolerogenic to cause-significant allergens at the initial stage of treatment, and SLIT later has provided the safety in long-term use. From our point of view, this method can be interesting when conducting AIT in children with AD, but for sure it is necessary to carry out the randomized studies to confirm its efficacy.

## Conclusion

The important advantages of AIT are: specific allergens and patients can be identified by suitable diagnostics; it is disease-modifying; it has long-lasting effect even after discontinuation; is more effective than pharmacotherapy. However, therapeutic utilization remains at low level due to poor compliance; inconvenient dosing; therapy with wild type allergen prone to have side effects.

The literature contains very limited evidence regarding the use of AIT in AD in children that dictates the need for additional large-scale randomized clinical trials with modern allergen and standardized protocols, the results of which will not only help to identify short-term and long-term protection from AD, but also to develop the guidelines and position papers regulating the use of AIT in children considering their peculiarities.

Most of the researches using AIT in AD in children are usually conducted in open uncontrolled trials or represent a series of reports on individual cases. These studies are difficult to compare, because there have been used the allergens from different pharmaceutical companies, doses and schemes of which, in this connection, are difficult to compare.

Using SLIT in AD is under study and more evidences are needed to support its clinical use, especially using SLIT in HDM allergens. Considering the excellent safety profile, the ability to influence on allergic inflammation, SLIT has huge potential to become a candidate for pathogenetic treatment of AD.

AIT, according to experts [12, 17, 19], has the following main unmet needs:

- Insufficient safety and efficacy;
- Insufficient profile of risk/benefit
- The lack of biomarkers to predict the course of the disease
- Optimal protocols (dose and frequency of administration)
- The Lack of general standard doses of allergens (aeroallergens in particular) in extracts for AIT (standardized extracts)
- Local and systemic reactions
- The Frequency of adverse effects and safety
- The definition of the duration and severity of the disease before treatment
- The minimum age of children for conducting AIT
- Difficulty in conducting large-scale, double-blind placebo-controlled, blind, randomized clinical trials in children
- Criteria for children selection to participate in randomized trials
- The efficacy and safety of AIT in patients not responding to pharmacotherapy
- Adherence to treatment
- The criteria for selection of patients for AIT
- The AIT optimization (the combined use of SCIT and SLIT and optimal terms for its conduction)
- Pharmaco-economic aspects (the high cost of AIT)
- Legal and ethical issues

**Improvements of current AIT required in four main aspects:**

- Efficacy: suppression of symptoms early and sustained
- Manufacturability: low cost and reproducible

During the manufacture of house dust mite extracts for diagnosis and immunotherapy in patients with AD it’s necessary to ensure the presence of all important mite allergens in the extracts. An improvement of the situation can only be expected from the use of defined recombinant allergens [15].

- Patient Convenience: fewer dose applications
- Safety: no anaphylaxis potential and no late phase reactions

However, the latest fundamental achievements in Immunology and Allergology have made a rapid breakthrough in the field of discovering the basic mechanisms of AIT and studying the major immune and not immune mechanisms of AD development, which are able not only to initiate the disease, but also affect on its duration and severity [34, 35, 42, 43, 49, 57, 83, 87].

In this regard the new strategies of AD treatment should include a comprehensive immunotherapy including the use of AIT and other biologically active agents able to interfere with pathophysiological processes [1–6, 44]. Use of the successes of modern molecular allergology, in particular, Molecular-based allergy diagnostics (Allergen Chip technology) will help to optimize and personalize the process of selecting the necessary allergens to determine the most appropriate vaccines for children considering the results of the allergen component diagnostics. Microchip diagnostics in pediatric Allergy is very important for conducting AIT: risk assessment and prognosis, improvement of the accuracy of diagnosis and cross-reactive sensitization in poly-sensitized patients, thereby improving the understanding of triggering allergens; identifying patients and triggering allergens for AIT [18]. Microarray diagnostics in pediatric allergy is the perfect tool for the diagnosis of IgE sensitization and allergic diseases in children.

The joint efforts of scientists and clinicians will certainly bring the benefits and will help the patients with AD to cope with their disease.

## Abbreviations

AD: atopic dermatitis
ADs: allergic diseases
AIT: allergen specific immunotherapy
AR: allergic rhinitis
BA: allergic bronchial asthma
BT: basic traditional therapy
ECP: eosinophil cationic protein
ELISA: enzyme-linked immunosorbent assay
HDMA: house dust mite allergens
SCIT: subcutaneous allergen specific immunotherapy
SLIT: sublingual allergen specific immunotherapy
