# Erratum to: Debates in allergy medicine: specific immunotherapy efficiency in children with atopic dermatitis

**DOI:** 10.1186/s40413-016-0128-x

**Published:** 2016-11-02

**Authors:** Tatiana A. Slavyanskaya, Vladislava V. Derkach, Revaz I. Sepiashvili

**Affiliations:** 1 People’s Friendship University of Russia, Moscow, Moscow region Russia; 2 Institute of Immunophysiology, Moscow, Russia; 3 Pacific State Medical University, Vladivostok, Russia

## Erratum

Unfortunately, the original version of this article [1] contained an error. The spelling of author name Tatiana A. Slavyanskaya was incorrect. The correct spelling can be found below.

Tatiana A. Slavyanskaya
